# Domestication and tameness: brain gene expression in red junglefowl selected for less fear of humans suggests effects on reproduction and immunology

**DOI:** 10.1098/rsos.160033

**Published:** 2016-08-03

**Authors:** Johan Bélteky, Beatrix Agnvall, Martin Johnsson, Dominic Wright, Per Jensen

**Affiliations:** AVIAN Behavioural Genomics and Physiology group, IFM Biology, Linköping University, 58183 Linköping, Sweden

**Keywords:** artificial selection, gene expression, microarray, chicken, fearfulness

## Abstract

The domestication of animals has generated a set of phenotypic modifications, affecting behaviour, appearance, physiology and reproduction, which are consistent across a range of species. We hypothesized that some of these phenotypes could have evolved because of genetic correlation to tameness, an essential trait for successful domestication. Starting from an outbred population of red junglefowl, ancestor of all domestic chickens, we selected birds for either high or low fear of humans for five generations. Birds from the fifth selected generation (S_5_) showed a divergent pattern of growth and reproduction, where low fear chickens grew larger and produced larger offspring. To examine underlying genetic mechanisms, we used microarrays to study gene expression in thalamus/hypothalamus, a brain region involved in fear and stress, in both the parental generation and the S_5_. While parents of the selection lines did not show any differentially expressed genes, there were a total of 33 genes with adjusted *p*-values below 0.1 in S_5_. These were mainly related to sperm-function, immunological functions, with only a few known to be relevant to behaviour. Hence, five generations of divergent selection for fear of humans produced changes in hypothalamic gene expression profiles related to pathways associated with male reproduction and to immunology. This may be linked to the effects seen on growth and size of offspring. These results support the hypothesis that domesticated phenotypes may evolve because of correlated effects related to reduced fear of humans.

## 1. Introduction

The domestication of animals represents an important event in the history of mankind, and has also been used ever since Darwin as a proof-of-principle in evolution [1]. During this process, animals have adapted to a life among humans, and have developed a set of similar traits, often referred to as the domesticated phenotype [2]. This includes changes in appearance and colour, reproduction, size and behaviour.

A recurring issue with respect to the domestication phenotype is whether it is the result of active human selection for each of the preferred traits independently, or whether the complex has developed as correlated responses to some central trait under selection early in history. For example, based on genetic mapping of differences between wild and domestic pigs, Rubin *et al.* [3] suggested that new colour phenotypes are a result of conscious human selection of preferred appearances, whereas Trut *et al.* [4] showed evidence that coloration may change as a secondary response to selection for increased tameness in experimentally domesticated foxes. The latter experiment is one of the most extensive in the field, and the results show that many of the commonly observed domesticated phenotypes, such as increased reproduction, colour and stress sensitivity changed when farm foxes were selected only for reduced fear of humans for a few generations. This suggests that tameness may in fact have been a driving factor behind large parts of the domesticated phenotype. Exactly how this works is yet unknown, but theories assuming pleiotropic effects have been proposed [5,6]. Among the more recent is the neural crest hypothesis, in which many of the traits associated with the domestic phenotype can be traced back to the role of neural crest cells (NCC) and deficits of NCCs during embryonic development.

Reduced fear is of course a first and necessary behavioural modification for successful domestication [7]. During the early history of domestication, animals able to thrive and reproduce in close proximity to humans would have a definite selective advantage, and any traits linked to this fearlessness would, therefore, potentially spread rapidly in the population. Investigating the nature of the links between different phenotypic traits may provide important insights into the evolution of phenotypic complexes (e.g. in species occupying similar ecological niches), and domestication offers a powerful model system for this. Using an inter-cross between tame and aggressive foxes from the previously mentioned selection experiment, Kukekova *et al.* [8] mapped a range of tameness traits to a small region on fox chromosome 12, homologous to a domestication-related locus in dogs. A similar approach revealed two tameness-related loci in experimentally selected rats, of which one overlapped a locus associated with adrenal weight [9]. Comparing gene expression differences in prefrontal cortex in a number of domesticated–wild pairs of mammals, Albert *et al.* found that variation in brain gene expression was considerably higher than nucleotide variation in DNA-sequence when comparing to RNA-seq data, and that only a few genes' transcription was affected in the same way in all domesticated species. For example, *SOX6* and *PROM1*, involved in brain development, were both upregulated in all domesticated variants [10]. Hence, there is a need for closer examination of the gene expression changes related to pathways affected by tameness and domestication.

The domestic chicken varieties of today all originate from the ancestral red junglefowl (*Gallus gallus*), native to the jungles of Southeast Asia [11,12]. Chickens were originally domesticated about 8000 years ago, and over the last hundred years, intense selection for growth and egg production has been carried out in a small number of commercial breeds. Modern chickens differ from their wild ancestors in a number of ways, typical for the domesticated phenotype; for example, with respect to size, colour, appearance, reproduction and behaviour [12–14]. In an ongoing experiment, we have selected populations of ancestral red junglefowl for reduced fear of humans during five generations and found that tamer birds became more dominant, laid larger eggs and produced larger offspring [15], and had a modified basal metabolic rate and increased feed conversion [16]. Genetic correlations between different traits suggest that, in this experimental population, major domestication-related traits appeared driven primarily by reduced fear of humans [17]. Among traits with a genetic correlation were the fear-of-human test, hatch weight, and movement and distance spent in periphery in an open field test at four weeks of age.

In this study, we explore the possible genetic mechanisms underlying the evolution of the observed suite of behavioural and reproductive traits associated with increased tameness in red junglefowl. Focusing on the hypothalamus, a central brain region coordinating fear and stress responses as part of the HPA-axis and regulator of the fight-or-flight response, the aim of the experiment is to assess genes and pathways that have changed their expression as a consequence of this selection.

## 2. Material and methods

### 2.1. Animals and sampling

Starting from an outbred laboratory population, we bred red junglefowl during five generations for increased versus reduced tameness. The breeding scheme and housing conditions have been described in detail elsewhere [15,17]. Briefly, birds bred and maintained with similar experiences of humans were selected in each generation based on divergent scores (high versus low fear) in a standardized fear-of-human test, with an unselected control population. The test was performed at 12 weeks of age for each generation, and involved a test person approaching a single bird in a 100 × 300 × 210 cm arena at certain steps over a 3 min time span. Over the duration of the test, fear scores were assigned to each bird based on a standardized ethogram. Each generation was maintained at about 50 birds per selection line, from 5 to 10 families per generation and selection line. The birds were hatched and reared under standardized conditions in mixed groups. They were weighed at hatch, and when 112 and 200 days old, and in addition, the weights of the offspring from the 5th generation were recorded.

For gene expression analysis, we studied animals from the parental (outbred and unselected) generation (P_0_) and from the fifth selected generation (S_5_). From the parental generation, we included only birds which were used as parents for the selection lines, i.e. those with extreme scores in the behavioural test. From S_5_, birds for inclusion in the analysis were randomly chosen from each selection line. In total, 24 individuals (four female and four male P_0_; three males and three females from the high selection line and three males and three females from the low selection line, and two females and two males from the unselected line in S_5_) were killed at the age of 350 days by rapid decapitation. Each brain was immediately dissected into seven parts (telencephalon, diencephalon, cerebellum, thalamus/hypothalamus and mesencephalon/pons) and snap frozen in liquid nitrogen within ten minutes of decapitation. They were then stored at −80°C until further analysis. Further studies for the present experiments were conducted only on the thalamus/hypothalamus part of the brain after dissection and removal of surrounding tissue.

### 2.2. Sample preparations

Following thawing, RNA was isolated from all samples and converted into double-stranded cDNA. A microarray analysis was performed on each of the 24 samples. One array failed quality control and was subsequently removed from the analysis.

RNA was extracted from thalamus/hypothalamus using an Allprep RNA/DNA kit (Qiagen) following the manufacturer's instructions. In short, approximately 20–30 mg tissue was homogenized with 600 µl Buffer RTL Plus using FastPrep®-24 (MP Biomedicals). RNA was separated from DNA using an AllPrep DNA spin column, and the filtered RNA was mixed with 150 µl chloroform, 80 µl Proteinase K and 350 µl 100% ethanol before being transferred to an RNeasy spin column. While bound to the column, RNA was cleaned through centrifugations with RPE Buffer, DNase I, Buffer FRN and pure ethanol before eluting the RNA into a 1.5 ml microcentrifuge tube using 30 µl of RNase-free water. The quantity of RNA samples were measured using a NanoDrop® ND-2000c (Thermo Scientific) followed by quality control with a Bioanalyzer® instrument (Agilent Technologies).

Samples were then treated once more with DNase I (Thermo Scientific) before synthesis of the first-strand cDNA using a Maxima H Minus First-Strand cDNA Synthesis Kit (Thermo Scientific), according to the protocol of the supplier. In short, DNase-treated template RNA was mixed with 10 mM dNTP mix, oligo(dT)18 primers and water during the annealing process. This step was followed by the addition of Maxima H Minus Enzyme Mix and buffer, then incubation at 50°C for 30 min. The second strand was synthesized by adding 30 U DNA Polymerase I, 1 U RNase H, 8 µl 10× reaction buffer and 68.8 µl water to each reaction, followed by incubation at 15°C for 2 h. After the incubation 12.5 U of T4 DNA polymerase was added to each reaction followed by further incubation at 15°C for 5 min before termination of the reaction with 5 µl 0.5 M EDTA (pH 8.0). Samples were purified through phenol/chloroform extraction and precipitated in 22 µl water, yielding sample concentrations at slightly above 100 ng µl^−1^. The dscDNA quality was controlled using the Bioanalyzer® (Agilent Technologies) and a GeneRuler 100 bp DNA Ladder (Thermo Scientific).

### 2.3. Gene expression analysis

All cDNA samples were labelled with Cy3 Random Nonamers using the NimbleGen One-Color DNA Labeling Kit (Roche NimbleGen), according to the protocol provided by the manufacturer. The Cy3-labelled cDNA samples were hybridized to NimbleGen chicken gene expression 12 × 135 k custom brain arrays (A-MTAB-542)(Roche NimbleGen). The custom chicken array is designed for brain gene expression with 60-mer oligonucleotide probes designed from Ensembl, RefSeq and EST sequences from the chicken. After washing of the slides, the arrays were scanned with an MS 200 Microarray Scanner (Roche NimbleGen).

### 2.4. Quantitative PCR

For four of the differentially expressed (DE) genes identified in the microarray, we performed qPCR amplifications to verify the results by an independent method. qPCR runs were performed with Maxima SYBR Green qPCR mastermix (Thermo Scientific) in 10 µl reactions, on Lightcycler 480 (Roche Applied Science). The primers were tested on and run on the following protocol: 5 min 95°C activation, 45 cycles of 10 s 95°C melting, 20 s 55°C annealing and 30 s 72°C elongation, followed by a melting of the product with temperatures increasing from 72 to 95°C. The crossing point values were normalized to reference genes TATA box binding protein and RNA polymerase II subunit C1 (Pol II), and the relative expression differences were calculated according to the method described by Pfaffl [18]. The genes and the primer sequences are detailed in electronic supplementary material, table S2.

### 2.5. Statistical analysis

Weight data were tested for normality with the Anderson–Darling test and residuals found to be sufficiently normally distributed for parametric analysis. They were, therefore, analysed with general linear models, using a repeated measures design for growth data. The model included sex, selection line and sex-selection interaction as main factors. The weight data analysis was performed using SPSS v. 23.

Microarray data were collected with the provided MS 200 Data Collection Software and normalized with the Robust Multiarray Average method using Deva software v. 1.2.2 (NimbleGen). The gene expression data were quality checked and analysed using R (http://www.r-project.org) and the Bioconductor packages (http://www.bioconductor.org). In short, microarray expression data were checked by comparing arrays with each other via logarithmic box plots of expression signals, and also by performing principal component analysis of the arrays. One sample was discarded after failing quality control of raw expression data. To find DE genes, a linear model approach was used with the *limma* package for R [19]. To adjust for multiple testing, Benjamini–Hochberg adjusted *p*-values were applied in order to determine statistical significance. The qPCR results were analysed by Student's *t*-test for the relative expression values between strains. Cluster analysis and heat maps were performed using the hierarchical clustering function with the Genesis software v. 1.7.6 [20]. Probe IDs were converted to ensemble gene IDs through the Database for Annotation, Visualization and Integrated discovery (DAVID), (https://david.ncifcrf.gov/), and then matched to the *G. gallus* array using the Manteia web tool (manteia.igbmc.fr) for subsequent functional analysis.

## 3. Results

**Figure 1.**
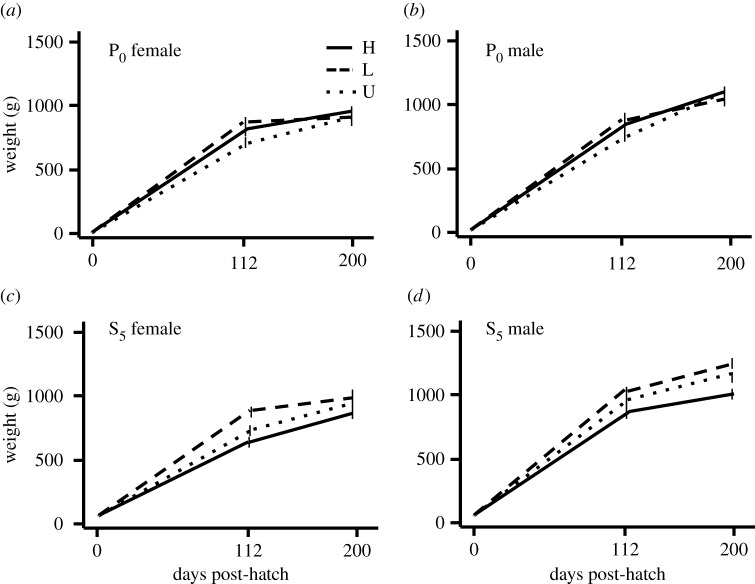
Growth curves for males and females from the parental generation (P_0_) and the fifth selected generation (S_5_). In P_0_, data show growth for the birds used as parents for generating the first selected generation of high (H) and low (L) fear of humans and the unselected birds (U). In S_5_, data show growth for all birds in each selection line. Mean values and standard deviations are given.

In the parental generation (P_0_) there were no significant differences in growth between the birds used as parents for the selection lines (figure 1*a*,*b*) (*F*_2,63_ = 2.2; *p* > 0.05). However, in S_5_, the low fear group grew significantly larger than the high fear group, with the unselected group showing intermediate weight development (figure 1*c*,*d*) (*F*_2,100_ = 44.2; *p* < 0.001). Furthermore, offspring of low fear birds from the S_5_ generation weighed significantly more than those of high fear birds (mean and s.e.m.; high fear: 22.9 ± 0.7; low fear: 26.4 ± 0.5; unselected: 26.3 ± 0.7; *F*_2,61_ = 7.2; *p* = 0.02).

There were no significantly DE genes between P_0_ birds assigned to the high and low fear strains. Hence, in the parental generation, gene expression profiles were similar in the birds which were chosen (based on the fear-of-human test) as parents for either the high or low fear selection strains. To further check that we had not unintentionally selected parental genes with different gene expression profiles, the 100 most DE genes sorted by *p*-value from generations P_0_ and S_5_ were compared with each other. The test showed no overlap of gene expression differences, so none of the top 100 DE genes in the parental generation was found among the top 100 in S_5_.

Comparing gene expression between either the high or low fear strains on one side and the unselected strain on the other in the S_5_ generation showed few significant expression differences between the groups when adjusting for multiple comparisons. Four probes in total were significantly DE in the two comparisons. In high fear versus unselected, *SPAG4* and *ENSGALT00000031518* (uncharacterized transcript) were highly expressed in the high fear strain, with *ENSGALT00000031518* following the same pattern in the low fear group but without reaching significance, therefore, not seemingly being strain specific. In the low fear strain, *MR1* and *ENSGALT00000009357*, both MHC-related transcripts, were both downregulated compared with the unselected group.

However, comparing the high versus low fear selection lines directly, 21 probes were significantly different at FDR-adjusted *p* < 0.05, and another 12 reached adjusted *p*-values below 0.1 (table 1). In the comparisons, positive fold change indicates higher expression in the high fear strain. The genes associated with the significantly different probes were predominantly related to male reproductive physiology (sperm function) both in the female and male brains, and with immunological functions, and only few annotated with terms relating to behaviour. For the additional 12 probes, there were several genes associated with signal transduction and/or regulation of transcription, and some of these have previously been associated with fear memory.

**Table 1.** Significantly differentially expressed genes comparing the high and low fear-of-human selection lines. Twenty-one genes were significantly DE when adjusting for multiple comparisons, and another 12 genes at *p* < 0.1 (below the line). The table gives the name of the gene where the annotation is known, its position (chromosome and start position in base pairs), the log fold change (FC) and the unadjusted as well as adjusted *p*-values.

gene	chromosome	start (bp)	log FC	*p* (unadj)	adj *p*
*MAEA*	4	87 576 507	−2.52067	1.12 × 10^−07^	0.001235
*MR1*	16	282 485	1.44795	6.16 × 10^−08^	0.001235
*ENSGALG00000024021*	22	3 859 032	3.847983	2.97 × 10^−07^	0.002178
*SFRP3*	7	2 296 056	1.46275	4.46 × 10^−07^	0.002264
*SPAG4*	25	691 949	4.384367	5.15 × 10^−07^	0.002264
*ENSGALG00000024195*	25	708 870	3.711317	1.64 × 10^−06^	0.004501
*ENSGALG00000026514*	16_random	48 484	1.421333	1.36 × 10^−06^	0.004501
*ANKRD1*	6	20 737 641	1.38885	2.31 × 10^−06^	0.005631
*ENSGALG00000016237*	1	115 815 831	1.922733	4.1 × 10^−06^	0.009019
*ROPN1 L*	2	80 353 846	−2.64702	5.89 × 10^−06^	0.010787
*MED22*	17	7 531 578	1.33565	5.62 × 10^−06^	0.010787
*GNB3*	1	80 401 456	1.01115	9.32 × 10^−06^	0.015762
*LOC771245*	22	3 858 483	2.43715	1.15 × 10^−05^	0.018056
*ERP27*	1	49 871 318	1.39875	1.83 × 10^−05^	0.025144
*GPRC5A*	1	50 100 860	2.050817	2.33 × 10^−05^	0.030124
*LOC418554*	1	113 884 100	−1.70378	3.47 × 10^−05^	0.042371
*RFT1*	12	1 136 949	−0.75372	4.46 × 10^−05^	0.044606
*MR1*	16_random	25 465	2.578417	4.28 × 10^−05^	0.044606
*C20orf134*	Z	44 144 955	−0.72155	4.34 × 10^−05^	0.044606
*RAB17*	7	4 751 928	1.080733	5.26 × 10^−05^	0.046384
*Q9DE41*	9	17 243 266	0.775517	5.03 × 10^−05^	0.046384
*ENSGALG00000003372*	8	5 281 952	1.082367	6.13 × 10^−05^	0.051828
*LOC417068*	16_random	11 985	1.341117	7.13 × 10^−05^	0.058066
*SAG*	9	2 143 184	1.476783	7.57 × 10^−05^	0.059414
*DPF3*	5	28 669 152	−1.25232	8.08 × 10^−05^	0.061243
*ITGB6*	7	23 382 082	−2.03637	8.44 × 10^−05^	0.061846
*PRL*	2	59 724 575	−0.77907	9.71 × 10^−05^	0.067596
*BRP44*	1	94 758 529	−1.42492	0.000107	0.071468
*CCDC58*	1	79 898 633	0.93905	0.000128	0.078171
*LOC422458*	4	31 764 144	−1.25643	0.000122	0.078171
*DYNLRB2*	11	16 668 352	−0.85143	0.000126	0.078171
*LOC420555*	2	22 624 460	0.996667	0.000146	0.086826
*GABRB1*	4	68 753 087	−1.14707	0.000166	0.095797

Comparisons between high and low fear lines for the sexes separately revealed five significantly differently expressed genes after FDR adjustment in females and 11 in the males, with four of the genes found in both sexes. Out of the total 16 significant genes, four were sex specific with *GNG11* found only in females and *POPDC3*, *ALDH5A1* and *LOC769497* in males. For further analyses, only grouped data was used to look for treatment effects between the two selected populations.

**Figure 2.**
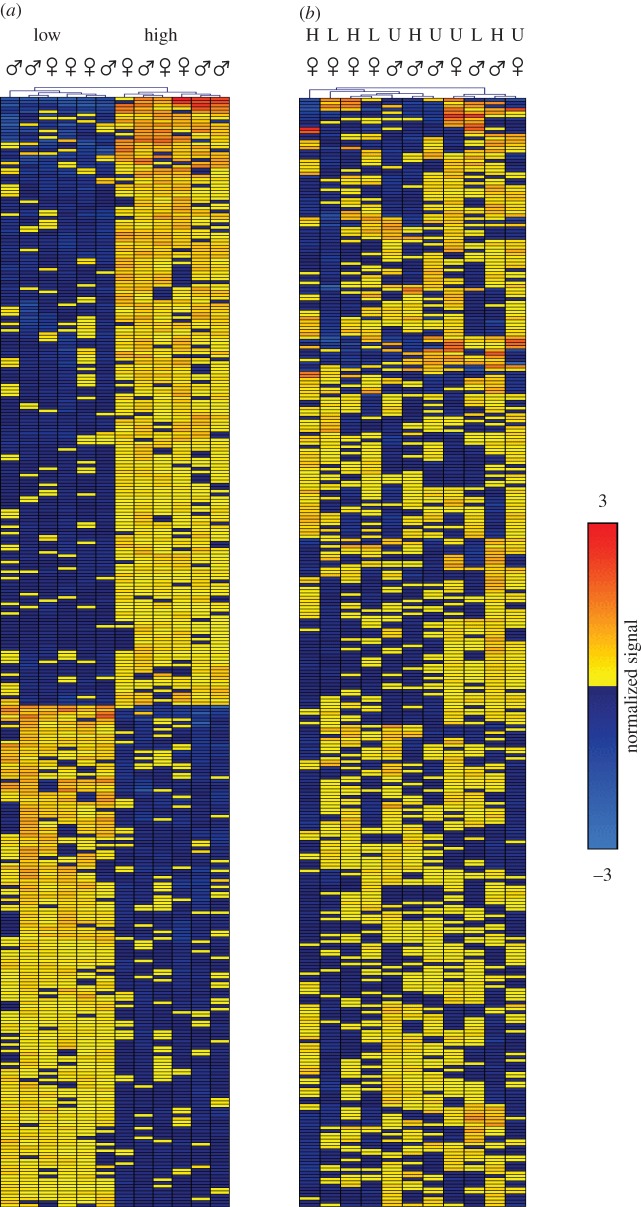
Heat maps for the relative expression levels of 344 genes with a differential expression reaching an unadjusted *p*-value of 0.01. Each column represents one bird and rows represent each of the 344 genes. The heat maps are generated using complete linkage hierarchical cluster analysis. (*a*) In generation S_5_, the high and low selection lines separate into two distinct clades based on the selection treatment. (*b*) In the P_0_ and unselected S_5_ lines, no clustering occurs either by treatment or sex. H, high; L, low; U, unselected.

To further explore gene expression changes, we generated heat maps of the top 344 DE probes in S_5_, based on a cut-off at *p* < 0.01, and disregarding corrections for multiple testing. The expression differences clustered clearly into two groups, separating the high and the low fear strains (figure 2), while a similar heat map for P_0_ shows no clustering into selection groups, emphasizing the fact that the fear-related expression differences were not present in the birds selected as parents for the selection lines. The full list of the probes and genes included in this analysis can be found in electronic supplementary material, table S1.

**Figure 3.**
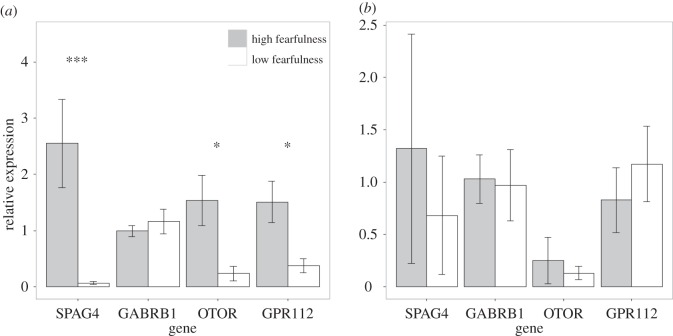
Quantitative PCR verification of gene expression of genes *SPAG4*, *GABRB1*, *OTOR* and *GPR112* in high and low lines in (*a*) the fifth selected generation (S_5_) and (*b*) parental generation (P_0_). Data are shown as means + s.e. (***p < 0.001, *p < 0.05).

From the top list, we arbitrarily selected four genes with significant DE between high and low fear strains in the S_5_ generation, in order to verify the expression differences by means of qPCR amplification. Three out of four genes showed significant DE patterns between high and low fear animals also with this method (figure 3*a*). The same genes were also tested with the same method on the animals from the parental generation, and in this case, none of them were DE (figure 3*b*).

To analyse gene ontology (GO) terms related to the gene expression changes, a GO analysis was carried out on the 344 genes displayed in figure 2 (comparing high versus low fear strains). Using a *p*-value cut-off of 0.1 (Benjamini–Hochberg procedure), we found an enrichment of 14 different terms in the dataset (table 2). All GO terms were associated with cellular components, and out of the 14 terms four *proton-transporting* terms are all variations of terms based on the same few genes included. These terms are linked to ATP synthase, the energy-creating process in the mitochondrial membrane.

**Table 2.** Gene ontology (GO) terms associated with the top 344 differentially expressed genes (listed in the electronic supplementary material, table S1). GO terms were selected based on a Benjamini–Hochberg procedure with *p*-value cut-off at 0.1.

GO ID	GO term
GO:0045263	proton-transporting ATP synthase complex, coupling factor *F*(*o*)
GO:0043229	intracellular organelle
GO:0012505	endomembrane system
GO:0005753	mitochondrial proton-transporting ATP synthase complex
GO:0043231	intracellular membrane-bounded organelle
GO:0045259	proton-transporting ATP synthase complex
GO:0044424	intracellular part
GO:0005622	intracellular
GO:0033061	DNA recombinase mediator complex
GO:0044444	cytoplasmic part
GO:0043227	membrane-bounded organelle
GO:0032398	MHC class Ib protein complex
GO:0000276	mitochondrial proton-transporting ATP synthase complex, coupling factor *F*(*o*)
GO:0043226	organelle

## 4. Discussion

Our results show that five generations of divergent selection for fear of humans in red junglefowl, ancestors of domesticated chickens, produced a change in hypothalamic gene expression profiles. Few DE genes were involved in pathways associated with fear and stress. Rather, the main effects were on pathways related to male reproduction and to immunology. This may be linked to the effects seen on growth and size of offspring, where low fear birds from the fifth selected generation were larger and produced larger offspring. These results corroborate earlier phenotypic studies of the same animals, showing considerable correlated effects of selection on reproduction, health, growth and behaviour. Although the mechanisms remain unclear, it appears that increased tameness, a prerequisite for successful early domestication, produced correlated modifications of brain gene expression patterns, possibly connected to phenotypic modifications not intentionally selected for.

The overarching aim of the present project was to increase the understanding of how domestication phenotypes may possibly have evolved as correlated traits to an initial decrease in fear towards humans, which was a central prerequisite for successful domestication. Previously, genetic correlations were found between the selected trait ‘fear of humans’ and other non-intentionally selected behaviours (e.g. foraging), and significant heritabilities were observed for both the selected trait and e.g. hatch weight, indicating substantial genetic contributions [17]. Furthermore, the birds from the low fear strain were more socially dominant, grew faster, laid larger eggs and produced larger offspring and had a better plumage condition, indicating less exposure to feather-pecking [15], and they showed a higher basal metabolism and a more efficient feed conversion [16]. Taken together, the phenotypic findings suggest that a domesticated phenotype can evolve in a few generations of intentional selection for only reduced fear of humans. Hence, the present experiment aimed at disclosing some of the genetic mechanisms involved in the process.

Starting from an outbred parental generation, parental birds were selected based on their scores in a standardized fear-of-human test [17]. Although the weight of the selected parents did not differ significantly, birds from the S_5_ generation grew significantly larger and produced larger offspring. This correlated response could be a secondary effect of the previously found effects on social dominance and feed efficiency [16], or be an effect of correlated modifications in gene expression profiles. These explanations are of course not mutually exclusive. It is remarkable that two of the DE genes (*LLPH* and *HELB*) are located centrally in a previously observed QTL region associated with growth in chickens. This QTL explained up to 20% of the variation in body weight in an F2-inter-cross between red junglefowl and domesticated white leghorn [21], and is also connected to several behavioural traits relevant from a domestication perspective [22].

We found no DE genes when comparing the parents of the selection lines. Hence, there were no indications that we unintentionally selected birds with divergent gene expression in the parental generation, and the risk is, therefore, small that our findings are mainly a result of genetic drift.

In the S_5_ generation, the selection lines differed significantly from each other on a number of genes, although only a few of them showed any differences when separately compared to the unselected birds. Since differences between selected and unselected would be expected to be rather subtle, compared with those between the two selection lines, these may be difficult to detect with the relatively low sample size used for the microarrays.

Surprisingly, the GO-analysis showed few obvious connections to processes related to stress or fear. Rather, most GO-terms were related to cellular components, with uncertain relationships to the selected behaviour. Responses to stress and fear differ largely between male and female chickens [23], and it may, therefore, not be surprising that the sexes do react differently to the selection imposed in the present experiment.

Considering the total of 21 probes, which were DE at a significance cut-off of *p* < 0.01, none of the genes were obviously related to the fear response selected for. In both sexes, the most significant genes were related to sperm biology and the rest mostly to immunological processes. It is quite likely that the sperm-related genes actually have a non-sperm-related function when expressed in the hypothalamus. However, it is interesting that two main phenotypic responses correlated to reduced fear in this population are increased growth and reproductive potential (larger offspring), which may perhaps be related to the gene expression pattern observed. Immunology-related genes tend to change expression levels as a response to stress [24], and this may be a contributing explanation for the present observations. Expanding the gene list to include those with adjusted *p*-values up to 0.1 added 12 more probes, with several genes associated with transcription factor regulation and signal transduction, among them *DPF3*, *PRL* and *GABRB1*.

The gene *DPF3* (D4 zinc and double PHD finger family 3) codes for a part of a transcription regulating protein that binds acetylated histones, with specificity to H3K14ac, known to be affected by stress [25,26]. *PRL* (prolactin) is a peptide hormone with many functions, including homeostatic and immune system regulation, and anxiolytic effects by inhibition of the HPA-axis activity. Prolactin is also expressed in the hypothalamus, and mediates behavioural adaptations related to social behaviour and care of offspring [27,28]. *GABRB1* (gamma-aminobutyric acid (GABA) A receptor subunit beta 1) is part of a receptor for the inhibitory neurotransmitter GABA, which plays an important role in the central nervous system. GABA A receptor expression changes have been linked to several psychiatric disorders, and in rats stress decreases hypothalamic beta subunit expression in a pathway important for stress-induced glucocorticoid secretion [29,30]. It is also interesting that a related subunit (GABRB2) has recently been shown to be strongly related to chicken anxiety behaviour [31] and the GABA-signalling pathway has been found to be clearly linked to domestication effects on stress in chickens [32].

A previous study in red junglefowl showed that DE genes related to fearfulness were largely associated with immune reactions [33]. Only one of the genes was also detected in the present experiment (*GSTK1*). Out of a short list of genes consistently DE in frontal cortex of a number of domesticated mammals [10], none was present on our top 344 lists. It is also of interest to compare the present results to those of an extensive analysis of selective sweeps associated with chicken domestication. Out of the 344 genes included in figure 2, 12 were located in selective sweep regions previously associated with chicken domestication [34], but none of the 17 significant genes were so. The genes overlapping with previous studies are presented in table 3.

**Table 3.** Differentially expressed genes overlapping with previous studies. Genes from the 344 most differentially expressed genes (listed in electronic supplementary material, table S1) comparing high and low fear-of-human lines were compared with genomic regions described in previous studies examining effects of fear and domestication. Growth1: growth-related QTL region on chromosome one, study by Carlborg and co-workers [21]; Fearfulness: study by Jöngren *et al.* [33]; Sweep: sweep regions related to chicken domestication as identified by Rubin *et al.* [34]; Domestication: differentially expressed genes between red junglefowl and white leghorn, study by Nätt *et al.* [24]. The table gives the gene (or the Ensembl transcript ID where the annotation is not known), the log fold change (FC), the *p*-value of the differential expression, the position of the gene (chromosome and start position) and the study with which it overlaps.

gene	log FC	*p*-value	chromosome	start (bp)	overlap
*LLPH*	0.374867	0.005501	1	36 238 092	growth1
*HELB*	−0.52582	0.00206	1	36 284 702	growth1
*GSTK1*	0.4637	0.005857	1	80 911 430	fearfulness
*IL1RL1*	0.885067	0.006408	1	138 087 257	sweep
*Q2AB82*	−1.05893	0.006573	1	195 761 188	sweep
*ENSGALT00000037406*	−0.72065	0.00079	2	54 566 872	sweep
*POPDC3*	−1.2191	0.002184	3	71 505 286	sweep
*ARFIP1*	−1.351	0.00643	4	35 021 774	domestication
*ENSGALT00000038460*	−0.67343	0.001688	4	35 304 273	sweep
*SLC25A21*	0.676417	0.00019	5	39 355 924	sweep
*ENSGALT00000028865*	0.520817	0.007059	7	6 796 343	sweep
*SNORA46*	−0.52145	0.004122	11	1 409 703	sweep
*BEAN*	0.430	0.00281	11	12 395 560	domestication
*STC2*	0.801533	0.004249	13	9 178 952	sweep
*GCNT7*	0.797	0.00706	20	11 943 466	domestication
*APITD1*	0.717817	0.00068	21	3 721 279	sweep
*SNORA55*	−0.42767	0.004629	23	5 736 022	sweep

It remains an open question whether the domesticated phenotype is a result of correlated responses to a single key trait, such as tameness, or whether each aspect of the phenotype is a result of independent human selection. However, the present results corroborate previous findings from foxes and chickens, showing that intentional selection based solely on reduced fear of humans may affect a wide range of seemingly unrelated traits [4,16]. Our gene expression results indicate that immunological and reproductive processes were affected by the selection. Hence, strong genetic responses to increased tameness were related to traits not intentionally selected for. This could indicate that correlated responses are important driving factors underlying the evolution of the domesticated phenotype.

After only five generations of selection from an outbred population, few traits should be considered fixed, especially in a population were inbreeding is avoided. Previous studies on the experimental population, however, found significant genetic correlations between several traits [17], and in this study, we find that variation for transcripts under selection seem to decrease, as seen by the large number of significantly differently expressed genes in the fifth selected generation compared to the parental generation. Hence, we can regard the few generations studied here as representing the earliest phases of domestication of a species, and we suggest that the results indicate that tameness, a necessary initial trait of any domesticate, may have been driving other traits involved in the domesticated phenotype, presumably by correlated mechanisms at the genomic level.

In conclusion, five generations of divergent selection for fear of humans in red junglefowl, produced a marked change in growth, reproduction and hypothalamic gene expression profiles. The affected genes mainly related to male reproduction and to immunology. These results suggest that increased tameness caused correlated modifications of brain gene expression patterns, possibly connected to the phenotypic changes observed in the same animals.

## Supplementary Material

Table S1. Significantly differentially expressed genes comparing the high and low fear of human selection lines. A list of 344 genes were generated when setting cut off at P (unadj) < 0.01. The table gives the microarray Probe ID, Ensembl Gene ID and name of the gene where the annotation is known, its position (chromosome and start position in base pairs), the log fold change (FC), and the unadjusted as well as adjusted P-values

## Supplementary Material

Table S2. Primer sequences used in qPCR verification of probe expression in P0 and S5 birds. Bird weight data. Bird weights at hatch, at 112, and 200 days for parental generation P0 and selection generation S5

## Supplementary Material

Bird Weight data

## Data accessibility

The weight data supporting this article are available as the electronic supplementary material. Microarray data have been uploaded to Array Express (http://www.ebi.ac.uk/arrayexpress/) under accession E-MTAB-4113.
