# Supplementary material for: Domestication and tameness: brain gene expression in red junglefowl selected for less fear of humans suggests effects on reproduction and immunology
Source: R Soc Open Sci. 2016 Aug 3;3(8):160033. doi: 10.1098/rsos.160033 (PMC5108935; doi:10.1098/rsos.160033)
Supplement: Table S1. Significantly differentially expressed genes comparing the high and low fear of human selection lines. A list of 344 genes were generated when setting cut off at P (unadj) < 0.01. The table gives the microarray Probe ID, Ensembl Gene ID and name of the gene where the annotation is known, i [file rsos160033supp1.pdf]

Table S1. Significantly differentially expressed genes comparing the high and low fear of human selection lines. A list of 344 genes were generated when setting cut off at P (unadj) < 0.01. The table gives the microarray Probe ID, Ensembl Gene ID and name of the gene where the annotation is known, its position (chromosome and start position in base pairs), the log fold change (FC), and the unadjusted as well as adjusted P-values.

Probe ID	Ensembl Gene ID	Gene	Chromosome	Start (bp)	logFC	P (unadj)	Adj P
ENSGALT00000010457	ENSGALG00000006473	PLXNA4B	1	2328433	-0,451	0,00870	0,56166
NM_205373	ENSGALG00000006563	SEMA3D	1	8884475	-0,443	0,00627	0,51487
ENSGALT00000039838	-	LOC768413	1	10751119	-0,444	0,00632	0,51487
NM_001079501	ENSGALG00000019358	LLPH	1	36238092	0,375	0,00550	0,49259
ENSGALT00000016069	ENSGALG00000009885	HELB	1	36284702	-0,526	0,00206	0,33464
ENSGALT00000030666	ENSGALG00000019351	-	1	37534434	0,727	0,00413	0,44886
ENSGALT00000028667	ENSGALG00000017960	-	1	48519859	0,661	0,00178	0,31401
ENSGALT00000019172	ENSGALG00000011739	ERP27	1	49871318	1,399	1,83E-05	0,02514
ENSGALT00000019248	ENSGALG00000011796	GPRC5A	1	50100860	2,051	2,33E-05	0,03012
ENSGALT00000019256	ENSGALG00000011798	HEBP1	1	50125098	-0,536	0,00088	0,23840
ENSGALT00000019301	ENSGALG00000011826	PLBD1	1	50825364	1,030	0,00230	0,34651
ENSGALT00000030615	ENSGALG00000019324	-	1	51129461	1,109	0,00046	0,17601
ENSGALT00000030578	ENSGALG00000019306	-	1	54705220	-1,267	0,00029	0,13649
ENSGALT00000020963	ENSGALG00000012853	-	1	58872239	-0,856	0,00241	0,35764
ENSGALT00000037586	-	Q5ZHK2	1	59346971	-0,483	0,00782	0,54803
ENSGALT00000037394	-	O42508	1	69127480	0,492	0,00412	0,44886
ENSGALT00000022724	ENSGALG00000014031	LYRM5	1	69351518	0,806	0,00091	0,23902
ENSGALT00000023005	ENSGALG00000014228	SMC1B	1	72583739	0,674	0,00152	0,30823
ENSGALT00000015796	ENSGALG00000014551	TTC38	1	73576045	-0,646	0,00071	0,21964
ENSGALT00000030506	-	CDKN1A	1	74373976	0,620	0,00080	0,22846
ENSGALT00000027942	ENSGALG00000017287	FGF6	1	75426646	-0,803	0,00427	0,44886
ENSGALT00000030041	ENSGALG00000019041	NOBOX	1	76779851	0,745	0,00505	0,47513
NM_001037839	ENSGALG00000014362	CD86	1	79726247	-1,028	0,00118	0,27324
ENSGALT00000023287	ENSGALG00000014413	CCDC58	1	79898633	0,939	0,00013	0,07817
ENSGALT00000022680	ENSGALG00000013999	FBXO40	1	79996528	0,740	0,00216	0,33715
ENSGALT00000023410	-	GNB3	1	80401456	1,011	9,32E-06	0,01576
ENSGALT00000023632	ENSGALG00000014662	RBP5	1	80567864	0,701	0,00187	0,32019
ENSGALT00000023710	ENSGALG00000014708	GSTK1	1	80911430	0,464	0,00586	0,51087
ENSGALT00000023808	ENSGALG00000014759	STYK1	1	81511980	-0,688	0,00547	0,49259
ENSGALT00000036924	-	TBXT	1	87434768	-0,836	0,00296	0,39336
ENSGALT00000030433	-	CIP2A	1	90879004	-0,634	0,00931	0,56507
ENSGALT00000024793	ENSGALG00000015363	HJURP	1	90966985	1,484	0,00354	0,43493
ENSGALT00000036863	-	BRP44	1	94758529	-1,425	0,00011	0,07147
ENSGALT00000024917	ENSGALG00000015453	DUSP27	1	95142047	-0,807	0,00661	0,52640
NM_001079739	ENSGALG00000015474	CD80	1	95614907	-1,049	0,00169	0,30823
ENSGALT00000024972	ENSGALG00000015485	ACP6	1	95765792	0,693	0,00209	0,33464
ENSGALT00000025369	ENSGALG00000015730	PRSS7	1	102986518	1,506	0,00302	0,39685
ENSGALT00000025402	ENSGALG00000015751	ATP5J	1	105994389	0,950	0,00130	0,29436
ENSGALT00000025797	ENSGALG00000016012	KCNE1	1	109340338	-0,725	0,00975	0,57299
NM_001030797	ENSGALG00000016199	CRYAA	1	113199266	-1,371	0,00680	0,52980
ENSGALT00000026119	ENSGALG00000016202	HSF2BP	1	113489887	0,593	0,00985	0,57381
ENSGALT00000030335	-	LOC418554	1	113884100	-1,704	3,47E-05	0,04237
ENSGALT00000030324	ENSGALG00000016237	-	1	115815831	1,923	4,10E-06	0,00902
ENSGALT00000026665	ENSGALG00000016523	RS1	1	123954222	0,905	0,00626	0,51487
ENSGALT00000036617	ENSGALG00000016602	ARHGAP6	1	127872311	-0,370	0,00879	0,56166
NM_204372	ENSGALG00000016636	ARSH	1	132545115	0,415	0,00856	0,56146
ENSGALT00000026949	ENSGALG00000016698	SHOX	1	133851753	0,627	0,00616	0,51487
NM_001024590	ENSGALG00000016785	IL1RL1	1	138087257	0,885	0,00641	0,51758
ENSGALT00000027248	ENSGALG00000016868	NALCN	1	147732557	-0,444	0,00780	0,54803
ENSGALT00000027415	-	EPSTI1	1	170988337	-1,281	0,00106	0,25804
ENSGALT00000027435	ENSGALG00000016981	SPERT	1	172146860	0,833	0,00276	0,37534
NM_205199	-	LPAR6	1	173089200	-0,772	0,00170	0,30823
ENSGALT00000028962	ENSGALG00000018255	gga-mir-15a	1	173700575	-1,066	0,00042	0,16612
ENSGALT00000042118	ENSGALG00000025205	SNORD102	1	180618434	-0,770	0,00992	0,57381
ENSGALT00000030090	ENSGALG00000019075	-	1	181894711	-0,808	0,00167	0,30823
ENSGALT00000027769	ENSGALG00000019061	MMP1	1	186797475	1,230	0,00845	0,56086
ENSGALT00000036395	-	Q2AB82	1	195761188	-1,059	0,00657	0,52549
NM_213577	ENSGALG00000017267	THRSP	1	197836615	1,251	0,00256	0,36586
ENSGALT00000036355	ENSGALG00000022696	KCNE3	1	200810372	1,154	0,00065	0,21273
ENSGALT00000015998	ENSGALG00000005282	NKTR	2	1831407	-0,675	0,00165	0,30823
ENSGALT00000008484	ENSGALG00000005292	KLHL40	2	1916552	0,896	0,00130	0,29436
ENSGALT00000031307	ENSGALG00000019701	-	2	2179085	-0,924	0,00519	0,48050

ENSGALT00000039188	-	Q5ZKZ2	2	2200899	0,438	0,00537	0,49259
ENSGALT00000009688	ENSGALG00000006018	SLC22A13	2	4846659	-0,596	0,00278	0,37534
ENSGALT00000031243	ENSGALG00000019672	LOC420493	2	16137024	-1,181	0,00028	0,13512
ENSGALT00000012903	ENSGALG00000007943	NEBL	2	18040904	-0,741	0,00705	0,53152
ENSGALT00000015310	ENSGALG00000009399	GATAD1	2	22571156	0,377	0,00501	0,47473
ENSGALT00000015324	ENSGALG00000009407	LOC420553	2	22597633	-0,870	0,00033	0,14415
NM_001030974	ENSGALG00000009467	C2H7orf64	2	22624460	0,997	0,00015	0,08683
ENSGALT00000015493	-	GNG11	2	23264674	1,452	0,00915	0,56252
ENSGALT00000031205	-	Q5ZHJ9	2	24861124	-0,864	0,00051	0,17921
ENSGALT00000018740	ENSGALG00000011501	-	2	40746635	0,993	0,00497	0,47473
ENSGALT00000019484	ENSGALG00000011946	-	2	44128337	-0,663	0,00897	0,56252
ENSGALT00000019997	ENSGALG00000023050	-	2	45511846	-0,896	0,00945	0,56907
ENSGALT00000019739	ENSGALG00000012083	GPR141	2	46302954	-1,025	0,00425	0,44886
ENSGALT00000037485	ENSGALG00000023035	C3orf24	2	47557974	-0,546	0,00366	0,44242
ENSGALT00000020117	ENSGALG00000012309	LOC100858584	2	49942807	-0,868	0,00134	0,29811
ENSGALT00000037406	-	-	2	54566872	-0,721	0,00079	0,22846
ENSGALT00000037403	ENSGALG00000023008	LOC426177	2	54582587	-0,781	0,00215	0,33715
ENSGALT00000010402	ENSGALG00000022990	-	2	57275058	-0,609	0,00227	0,34651
ENSGALT00000020670	ENSGALG00000012663	ALDH5A1	2	58969224	-1,360	0,00422	0,44886
NM_205466	ENSGALG00000012671	PRL	2	59724575	-0,779	9,71E-05	0,06760
ENSGALT00000021239	ENSGALG00000013006	ROPN1L	2	80353846	-2,647	5,89E-06	0,01079
ENSGALT00000042131	ENSGALG00000025218	gga-mir-1681	2	96361703	-0,897	0,00306	0,39856
ENSGALT00000036912	ENSGALG00000022876	-	2	99280398	-0,580	0,00481	0,46623
ENSGALT00000024370	ENSGALG00000015104	HRH4	2	106554028	0,533	0,00711	0,53152
ENSGALT00000030896	-	Q5F3T5	2	116236062	-0,897	0,00332	0,41209
ENSGALT00000025032	ENSGALG00000015526	C8orf45	2	119619233	-0,743	0,00442	0,44952
ENSGALT00000025752	-	Q6V0P0	2	131075954	-0,639	0,00727	0,53679
ENSGALT00000025802	ENSGALG00000016016	OSR2	2	132808941	1,010	0,00702	0,53152
ENSGALT00000025824	-	RGS22	2	133439511	1,404	0,00468	0,46312
ENSGALT00000028783	ENSGALG00000018076	-	2	153830032	-0,836	0,00776	0,54803
ENSGALT00000022502	ENSGALG00000013875	FAM83H	2	154662706	-0,642	0,00483	0,46623
NM_204732	ENSGALG00000008732	OTOR	3	5584685	2,467	0,00162	0,30823
ENSGALT00000014515	ENSGALG00000008931	COMMD1	3	8903451	0,770	0,00048	0,17613
ENSGALT00000014511	-	TMEM17	3	9036575	0,831	0,00417	0,44886
NM_001079490	ENSGALG00000009093	PNO1	3	11182638	0,460	0,00452	0,45411
ENSGALT00000014952	ENSGALG00000009178	-	3	13171673	-0,654	0,00108	0,25913
ENSGALT00000015245	ENSGALG00000009360	CAPN2	3	18818136	-0,462	0,00906	0,56252
ENSGALT00000017249	ENSGALG00000010599	SULT6B1	3	34763544	0,445	0,00308	0,39856
ENSGALT00000017478	ENSGALG00000010741	MAP1LC3C	3	36967939	0,655	0,00870	0,56166
ENSGALT00000028630	ENSGALG00000017923	U1	3	40653244	-0,538	0,00263	0,36586
NM_001114081	ENSGALG00000011418	CCR6	3	44326680	0,903	0,00501	0,47473
ENSGALT00000031763	ENSGALG00000019961	C6orf192	3	58709807	0,393	0,00604	0,51487
ENSGALT00000023930	ENSGALG00000014823	LOC769246	3	61346649	0,571	0,00440	0,44952
NM_001001299	ENSGALG00000015410	POPDC3	3	71505286	-1,219	0,00218	0,33814
ENSGALT00000042480	ENSGALG00000025567	gga-mir-1686	3	78289225	-0,533	0,00819	0,54993
ENSGALT00000025725	-	C6orf57	3	85437845	1,184	0,00540	0,49259
ENSGALT00000037050	-	CO9A1	3	85586690	0,919	0,00799	0,54803
ENSGALT00000026224	ENSGALG00000016263	LOC100858320	3	88322424	-1,098	0,00162	0,30823
ENSGALT00000026225	-	LOC428640	3	88367930	-1,033	0,00297	0,39336
ENSGALT00000026230	ENSGALG00000016267	PHF3	3	88409177	-0,448	0,00266	0,36586
NM_001012880	ENSGALG00000016278	PRIM2	3	89520703	2,042	0,00316	0,40385
NM_206980	ENSGALG00000016316	GCM1	3	91112647	0,534	0,00709	0,53152
ENSGALT00000026524	ENSGALG00000016441	-	3	99381626	0,984	0,00261	0,36586
ENSGALT00000031591	ENSGALG00000019873	RDH14	3	103442251	0,442	0,00763	0,54803
ENSGALT00000020703	-	KIF13B	3	109136613	-0,655	0,00312	0,40056
NM_204650	ENSGALG00000016670	DEFB1	3	110267211	1,204	0,00789	0,54803
ENSGALT00000031538	-	GLL4	3	110276282	-0,790	0,00680	0,52980
ENSGALT00000006540	ENSGALG00000004113	LOC422147	4	1432944	0,588	0,00871	0,56166
ENSGALT00000010323	ENSGALG00000006393	GPR112	4	4315284	1,953	0,00256	0,36586
ENSGALT00000011597	ENSGALG00000007160	ARHGAP36	4	9586935	0,649	0,00746	0,54471
ENSGALT00000011832	ENSGALG00000007311	CLDN2	4	11034232	0,550	0,00568	0,49969
ENSGALT00000012180	ENSGALG00000007530	LOC428714	4	11396340	0,940	0,00209	0,33464
ENSGALT00000012638	ENSGALG00000007793	UPRT	4	12653329	0,585	0,00383	0,44759
ENSGALT00000032247	ENSGALG00000020233	LOC422458	4	31764144	-1,256	0,00012	0,07817
ENSGALT00000038470	-	ARFIP1	4	35021774	-1,351	0,00643	0,51758
ENSGALT00000038460	ENSGALG00000023351	-	4	35304273	-0,673	0,00169	0,30823
ENSGALT00000017168	ENSGALG00000010550	GIMD1	4	39781462	-0,645	0,00327	0,41108
ENSGALT00000017495	-	HAND2	4	44939186	0,602	0,00875	0,56166
NM_001001203	ENSGALG00000010864	EREG	4	46163378	0,789	0,00677	0,52980
ENSGALT00000018988	ENSGALG00000011623	ADAMTS3	4	52098559	-0,506	0,00889	0,56252

ENSGALT00000019554	ENSGALG00000011986	FABP2	4	56264371	-0,437	0,00967	0,57149
NM_001079759	ENSGALG00000012181	RRH	4	59684850	0,655	0,00850	0,56133
ENSGALT00000021879	-	PDLI3	4	62917937	0,914	0,00048	0,17613
ENSGALT00000022965	ENSGALG00000014200	GABRB1	4	68753087	-1,147	0,00017	0,09580
NM_204447	ENSGALG00000014501	FGFBP2	4	79507016	-0,884	0,00614	0,51487
ENSGALT00000037157	-	MAEA	4	87576507	-2,521	1,12E-07	0,00123
ENSGALT00000025359	ENSGALG00000015723	-	4	88180622	0,775	0,00020	0,10926
ENSGALT00000010345	ENSGALG00000006407	Q7T3M7	5	14153126	0,635	0,00775	0,54803
ENSGALT00000035359	ENSGALG00000021817	-	5	15852722	-0,574	0,00433	0,44886
ENSGALT00000032681	ENSGALG00000020485	-	5	17959253	-0,738	0,00320	0,40690
ENSGALT00000012190	ENSGALG00000007537	INCENP	5	18267636	0,552	0,00919	0,56252
ENSGALT00000038620	-	API5	5	20371044	-0,830	0,00740	0,54236
ENSGALT00000014202	ENSGALG00000008727	JMJD7-PLA2G4B	5	27300442	-0,815	0,00597	0,51487
ENSGALT00000014889	ENSGALG00000009145	STARD9	5	27998541	-0,541	0,00385	0,44822
ENSGALT00000014915	ENSGALG00000009156	CDAN1	5	28042117	-0,488	0,00075	0,22659
ENSGALT00000015230	ENSGALG00000009352	DPF3	5	28669152	-1,252	8,08E-05	0,06124
ENSGALT00000015283	ENSGALG00000009381	SIPA1L1	5	29210163	-0,401	0,00811	0,54993
NM_001197057	ENSGALG00000020449	COX16	5	29828587	0,547	0,00137	0,29811
NM_001031197	ENSGALG00000009421	SFRS5	5	30168433	-0,698	0,00832	0,55565
ENSGALT00000015453	ENSGALG00000009491	RAD51L1	5	31028909	0,577	0,00108	0,25913
ENSGALT00000015569	ENSGALG00000009562	PLEK2	5	31252027	1,524	0,00820	0,54993
ENSGALT00000032603	-	LOC428880	5	32373421	-0,856	0,00639	0,51758
NM_204978	ENSGALG00000009724	GREM1	5	32892433	-0,774	0,00335	0,41335
ENSGALT00000016457	ENSGALG00000010117	SLC25A21	5	39355924	0,676	0,00019	0,10713
ENSGALT00000016505	ENSGALG00000010154	GEMIN2	5	39989268	0,585	0,00626	0,51487
ENSGALT00000018168	ENSGALG00000011139	CCDC85C	5	50606598	-0,674	0,00901	0,56252
ENSGALT00000028573	ENSGALG00000017866	SNORA28	5	52733281	-0,637	0,00605	0,51487
ENSGALT00000019409	ENSGALG00000011893	SLC38A6	5	56814010	-0,477	0,00960	0,57041
ENSGALT00000002873	ENSGALG00000001854	LOC423605	6	1886044	1,032	0,00143	0,30171
ENSGALT00000041181	-	CLAT	6	3966956	0,606	0,00159	0,30823
ENSGALT00000003952	ENSGALG00000002507	MBL2	6	5578097	1,111	0,00136	0,29811
ENSGALT00000004324	ENSGALG00000002744	Q0ZM14	6	7469709	-0,963	0,00373	0,44549
ENSGALT00000005703	ENSGALG00000003603	LOC770890	6	10536007	0,715	0,00168	0,30823
ENSGALT00000007975	ENSGALG00000004973	DLG5	6	14312746	-1,051	0,00953	0,56907
ENSGALT00000040000	-	Q52P71	6	19079726	-0,572	0,00917	0,56252
NM_204405	ENSGALG00000006491	ANKRD1	6	20737641	1,389	2,31E-06	0,00563
ENSGALT00000010634	-	TMEM20	6	21361910	0,870	0,00720	0,53471
NM_204986	ENSGALG00000006626	PDE6C	6	21481667	-0,629	0,00878	0,56166
ENSGALT00000011349	ENSGALG00000007014	PYROXD2	6	23673992	-0,849	0,00229	0,34651
NM_001039305	ENSGALG00000007697	MGEA5	6	24104164	0,863	0,00632	0,51487
ENSGALT00000042170	ENSGALG00000025257	gga-mir-1674	6	24237813	-0,872	0,00544	0,49259
ENSGALT00000013657	ENSGALG00000008383	SFR1	6	25634354	-0,753	0,00882	0,56219
ENSGALT00000015025	ENSGALG00000009231	PNLIP	6	30327112	1,212	0,00147	0,30823
ENSGALT00000034022	ENSGALG00000002594	TFPI	7	1041837	-0,965	0,00264	0,36586
NM_204772	ENSGALG00000002763	FRZB	7	2296056	1,463	4,46E-07	0,00226
ENSGALT00000005620	ENSGALG00000003553	ABCA12	7	4317198	0,917	0,00568	0,49969
NM_001128496	ENSGALG00000026378	IFNL3	7	4546938	-0,464	0,00483	0,46623
ENSGALT00000006196	ENSGALG00000003899	RAB17	7	4751928	1,081	5,26E-05	0,04638
ENSGALT00000028865	ENSGALG00000018158	-	7	6796343	0,521	0,00706	0,53152
ENSGALT00000003467	ENSGALG00000002213	-	7	6868257	0,542	0,00619	0,51487
ENSGALT00000013740	-	FZD7	7	12741968	-1,344	0,00090	0,23902
NM_204135	ENSGALG00000008435	SUMO1	7	12830281	-0,691	0,00099	0,24970
ENSGALT00000042006	ENSGALG00000025093	SNORD70	7	12847697	1,317	0,00026	0,13448
ENSGALT00000013870	ENSGALG00000008516	CPO	7	13436148	0,399	0,00574	0,50277
NM_205311	ENSGALG00000008669	CD28	7	14540564	-1,194	0,00668	0,52837
ENSGALT00000014289	ENSGALG00000008786	NBEAL1	7	14806915	-0,547	0,00666	0,52837
ENSGALT00000014307	ENSGALG00000008803	LOC424111	7	14877303	1,071	0,00990	0,57381
NM_001195424	ENSGALG00000020793	UBE2E3	7	15667519	-0,915	0,00061	0,20712
ENSGALT00000038690	ENSGALG00000023418	-	7	17399442	0,479	0,00971	0,57221
NM_204816	ENSGALG00000009301	CHRNA1	7	17979759	-0,594	0,00081	0,22846
ENSGALT00000021609	ENSGALG00000013231	-	7	18993533	-0,639	0,00787	0,54803
NM_204207	ENSGALG00000009525	HAT1	7	19333876	0,473	0,00951	0,56907
ENSGALT00000015986	-	LOC424161	7	20057522	-0,685	0,00361	0,43789
ENSGALT00000016000	ENSGALG00000009841	FASTKD1	7	20108664	0,412	0,00782	0,54803
ENSGALT00000017341	ENSGALG00000010657	-	7	20177146	-0,956	0,00567	0,49969
ENSGALT00000038475	-	RDH5	7	20326319	-2,180	0,00038	0,15255
ENSGALT00000033140	ENSGALG00000020703	GRB14	7	21655865	0,629	0,00078	0,22846
ENSGALT00000018170	ENSGALG00000011141	ITGB6	7	23382082	-2,036	8,44E-05	0,06185
NM_001012918	ENSGALG00000011232	STK11IP	7	23619801	0,712	0,00388	0,44886
ENSGALT00000018322	ENSGALG00000011233	GJD3	7	23640259	-0,580	0,00949	0,56907

ENSGALT00000034240	ENSGALG00000021244	-	8	126428	-0,540	0,00624	0,51487
ENSGALT00000004839	ENSGALG00000003064	TADA1L	8	4344436	0,589	0,00163	0,30823
ENSGALT00000005331	ENSGALG00000003372	-	8	5281952	1,082	6,13E-05	0,05183
ENSGALT00000005810	ENSGALG00000003666	-	8	5908737	1,124	0,00032	0,14415
ENSGALT00000005817	ENSGALG00000003671	RGSL1	8	5922421	0,814	0,00243	0,35841
ENSGALT00000006927	ENSGALG00000004341	LOC424430	8	6914850	1,006	0,00210	0,33464
ENSGALT00000009470	ENSGALG00000005889	CCDC18	8	14675638	-0,507	0,00324	0,40944
ENSGALT00000010222	ENSGALG00000006322	LOC424523	8	16678068	-0,415	0,00692	0,53152
NM_001031281	ENSGALG00000010234	MMACHC	8	21848762	0,508	0,00381	0,44759
ENSGALT00000017079	ENSGALG00000010487	-	8	22729974	-0,837	0,00895	0,56252
ENSGALT00000017472	ENSGALG00000010736	DIO1	8	25857638	0,982	0,00620	0,51487
ENSGALT00000028882	ENSGALG00000018175		8	26034075	-0,514	0,00853	0,56133
ENSGALT00000032964	ENSGALG00000020614	TM2D1	8	28094837	0,508	0,00182	0,31478
ENSGALT00000041322	ENSGALG00000024414	-	9	1699226	-0,727	0,00075	0,22659
NM_001031300	ENSGALG00000001302	LOC424740	9	1878425	0,583	0,00960	0,57041
ENSGALT00000002309	ENSGALG00000001519	NEU2	9	2059175	0,932	0,00260	0,36586
ENSGALT00000002605	ENSGALG00000001696	SAG	9	2143184	1,477	7,57E-05	0,05941
ENSGALT00000041251	ENSGALG00000024385	-	9	3186431	0,592	0,00080	0,22846
ENSGALT00000010653	ENSGALG00000006600	A4GNT	9	5135556	0,876	0,00399	0,44886
ENSGALT00000010387	ENSGALG00000006436	AQP12	9	5671109	0,672	0,00097	0,24756
ENSGALT00000010372	ENSGALG00000006426	PAK2	9	5696674	-0,792	0,00439	0,44952
ENSGALT00000011695	ENSGALG00000007226	OSTN	9	14850757	1,254	0,00800	0,54803
ENSGALT00000014019	-	Q9DE41	9	17243266	0,776	5,03E-05	0,04638
ENSGALT00000014769	ENSGALG00000009077	-	9	19588026	-0,643	0,00483	0,46623
ENSGALT00000015314	ENSGALG00000009402	LRRC34	9	21484477	-0,466	0,00912	0,56252
ENSGALT00000015394	ENSGALG00000009458	LOC424998	9	21923408	-0,613	0,00863	0,56166
ENSGALT00000015499	-	LOC429161	9	23491582	0,651	0,00413	0,44886
ENSGALT00000039223	ENSGALG00000023582	-	9	23543733	0,725	0,00671	0,52874
ENSGALT00000015715	ENSGALG00000009651	LXN	9	24010707	0,535	0,00402	0,44886
ENSGALT00000016913	ENSGALG00000023498	-	9	25175606	-0,557	0,00807	0,54899
NM_001030392	ENSGALG00000025885	COR7A	10	109319	0,640	0,00086	0,23810
NM_205425	ENSGALG00000001318	CSK	10	1798042	0,876	0,00683	0,53081
ENSGALT00000002010	ENSGALG00000001318	CSK	10	1800247	0,781	0,00430	0,44886
ENSGALT00000040024	-	Q9PRY6	10	1809248	-0,634	0,00699	0,53152
ENSGALT00000004280	-	LOC415345	10	3697373	-0,513	0,00510	0,47536
ENSGALT00000006599	ENSGALG00000004150	MYO1E	10	7738765	-0,698	0,00963	0,57073
ENSGALT00000006734	-	LIPC	10	7968058	-0,946	0,00201	0,33418
ENSGALT00000007393	ENSGALG00000004641	MYO5C	10	10091529	-0,465	0,00778	0,54803
ENSGALT00000038917	-	Q5ZKJ3	10	11635337	0,486	0,00919	0,56252
ENSGALT00000009730	ENSGALG00000006041	BNC1	10	13007552	1,090	0,00180	0,31401
ENSGALT00000002005	-	Q5ZJF9	11	655781	-0,568	0,00911	0,56252
ENSGALT00000001062	-	LOC769608	11	1123147	0,487	0,00169	0,30823
ENSGALT00000028532	ENSGALG00000017825	SNORA46	11	1409703	-0,521	0,00412	0,44886
ENSGALT00000034267	ENSGALG00000021260	LOC415728	11	6551898	-0,905	0,00103	0,25373
ENSGALT00000008318	ENSGALG00000005178	CA7	11	12183227	1,951	0,00091	0,23902
ENSGALT00000042240	ENSGALG00000025327	gga-mir-1634	11	12200275	-1,132	0,00045	0,17489
ENSGALT00000033882	-	LOC769939	11	12312498	0,473	0,00495	0,47468
ENSGALT00000008438	ENSGALG00000005254	CKLF	11	12324317	0,593	0,00167	0,30823
NM_001141926	ENSGALG00000005266	BEAN	11	12395560	0,430	0,00281	0,37707
ENSGALT00000008677	ENSGALG00000005402	DYNLRB2	11	16668352	-0,851	0,00013	0,07817
ENSGALT00000023417	ENSGALG00000014506	-	11	20574569	-0,574	0,00236	0,35347
ENSGALT00000042215	ENSGALG00000025302	SNORD71	11	21753369	-0,665	0,00435	0,44886
NM_213580	ENSGALG00000001709	MSTN1	12	809638	0,641	0,00049	0,17801
ENSGALT00000003010	ENSGALG00000001945	RFT1	12	1136949	-0,754	4,46E-05	0,04461
ENSGALT00000006258	ENSGALG00000003927	-	12	1583003	0,586	0,00710	0,53152
ENSGALT00000003585	ENSGALG00000002283	MAPKAPK3	12	1748813	0,877	0,00162	0,30823
ENSGALT00000003843	ENSGALG00000002436	LOC101748064	12	2161883	0,977	0,00164	0,30823
NM_001001460	ENSGALG00000004974	PPARG	12	5069039	0,973	0,00789	0,54803
ENSGALT00000008038	ENSGALG00000005021	RAB43	12	5308554	1,401	0,00850	0,56133
ENSGALT00000036264	ENSGALG00000005332	CAC1D	12	7278350	-0,544	0,00922	0,56252
ENSGALT00000041402	ENSGALG00000024489	LOC416082	12	14259331	0,906	0,00704	0,53152
ENSGALT00000012074	ENSGALG00000007467	SLC25A26	12	14671082	0,473	0,00795	0,54803
ENSGALT00000034681	ENSGALG00000021471	IL17B	13	8360367	1,760	0,00208	0,33464
ENSGALT00000040996	ENSGALG00000002893	STC2	13	9178952	0,802	0,00425	0,44886
ENSGALT00000040541	-	Q5ZHW8	13	13218400	0,713	0,00261	0,36586
ENSGALT00000040457	-	HMD1	13	13322789	-0,649	0,00800	0,54803
ENSGALT00000009939	-	LOC416300	13	14686959	0,773	0,00193	0,32404
ENSGALT00000011489	ENSGALG00000007096	D0VX32	13	17654109	0,602	0,00032	0,14415
NM_204267	ENSGALG00000003217	LITAF	14	923094	0,611	0,00944	0,56907
ENSGALT00000008758	ENSGALG00000005458	RPL3L	14	6168193	0,426	0,00990	0,57381

ENSGALT00000034515	ENSGALG00000021386	LOC416390	14	14158026	0,916	0,00420	0,44886
ENSGALT00000033185	-	LOC770114	14	14403164	1,885	0,00038	0,15255
ENSGALT00000039277	ENSGALG00000023602	-	14	14612798	0,979	0,00035	0,14883
ENSGALT00000014982	ENSGALG00000009201	CCNF	14	15022912	-0,440	0,00359	0,43789
ENSGALT00000042285	ENSGALG00000025372	gga-mir-1636	15	4729959	-0,551	0,00935	0,56646
ENSGALT00000038541	ENSGALG00000005865	O13004	15	8105501	0,640	0,00434	0,44886
ENSGALT00000009756	ENSGALG00000021126	LOC416935	15	8283067	-1,123	0,00191	0,32301
ENSGALT00000010884	ENSGALG00000006728	SLC5A1	15	9050648	-0,731	0,00657	0,52549
ENSGALT00000011065	ENSGALG00000006838	LOC416959	15	9181103	0,758	0,00207	0,33464
ENSGALT00000011306	ENSGALG00000023311	-	15	9429328	-0,829	0,00407	0,44886
ENSGALT00000022037	ENSGALG00000024348	MR1	16	282485	1,448	6,16E-08	0,00123
ENSGALT00000009998	ENSGALG00000024340	LOC769497	16	345849	1,507	0,00021	0,11180
ENSGALT00000028239	ENSGALG00000004772	Q6X3Y9	16	351638	0,416	0,00752	0,54736
ENSGALT00000039339	ENSGALG00000003204	MED22	17	7531578	1,336	5,62E-06	0,01079
NM_001030679	-	DENND1A	17	9912496	-0,668	0,00473	0,46590
ENSGALT00000039546	-	Q90908	18	612846	0,856	0,00983	0,57381
ENSGALT00000004769	-	COX11	18	5839577	0,429	0,00445	0,45079
ENSGALT00000028755	ENSGALG00000018048	-	18	7403035	-0,562	0,00213	0,33715
NM_001031530	ENSGALG00000007386	LUC7L3	18	10087770	-0,568	0,00839	0,55883
NM_001045831	ENSGALG00000000956	GGCL1	19	355104	0,556	0,00520	0,48050
NM_001030714	ENSGALG00000002576	ASL2	19	4906156	0,441	0,00265	0,36586
ENSGALT00000039978	-	ARLY2	19	4906249	0,417	0,00435	0,44886
ENSGALT00000005549	-	LOC419164	20	3614084	1,342	0,00990	0,57381
ENSGALT00000033867	-	Q7ZZY5	20	9628695	0,694	0,00066	0,21273
ENSGALT00000012508	ENSGALG00000007717	GCNT7	20	11943466	0,797	0,00706	0,53152
NM_204979	ENSGALG00000012070	CYP24A1	20	12321164	0,602	0,00760	0,54740
NM_205253	ENSGALG00000008014	CEBPB	20	13766624	0,463	0,00924	0,56252
ENSGALT00000000797	ENSGALG00000000573	PER3	21	280881	-0,995	0,00458	0,45796
ENSGALT00000001006	ENSGALG00000000710	RNF207	21	597980	0,884	0,00371	0,44549
ENSGALT00000040047	-	H6PD	21	3284471	0,679	0,00138	0,29811
ENSGALT00000041394	ENSGALG00000024481	APITD1	21	3721279	0,718	0,00068	0,21662
ENSGALT00000029675	ENSGALG00000018791	LOC771245	22	3858483	2,437	1,15E-05	0,01806
ENSGALT00000031473	ENSGALG00000024021	-	22	3859032	3,848	2,97E-07	0,00218
ENSGALT00000028555	ENSGALG00000017848	SNORA55	23	5736022	-0,428	0,00463	0,46040
ENSGALT00000042615	ENSGALG00000025702	5S rRNA	24	1053187	-0,710	0,00229	0,34651
ENSGALT00000001863	ENSGALG00000024296	LOC419726	24	1453350	1,094	0,00631	0,51487
ENSGALT00000000600	ENSGALG00000000443	SPAG4	25	691949	4,384	5,15E-07	0,00226
ENSGALT00000040879	ENSGALG00000024193	-	25	714586	0,727	0,00508	0,47536
ENSGALT00000040807	ENSGALG00000027475	LOC430658	25	1023625	0,944	0,00914	0,56252
ENSGALT00000040796	-	LOC426916	25	1092350	0,757	0,00549	0,49259
ENSGALT00000014969	ENSGALG00000009190	-	25	1135097	-0,753	0,00103	0,25373
ENSGALT00000014968	ENSGALG00000024138	KRSC	25	1167623	-0,521	0,00755	0,54736
ENSGALT00000014945	ENSGALG00000018878	PRR9	25	1267265	-0,925	0,00305	0,39856
ENSGALT00000001237	ENSGALG00000000844	SLC26A8	26	134800	-0,409	0,00874	0,56166
ENSGALT00000040764	-	LAD1	26	776460	0,785	0,00179	0,31401
NM_001044644	ENSGALG00000000919	PIGR	26	2401255	1,266	0,00593	0,51487
ENSGALT00000009242	ENSGALG00000005756	-	27	262898	1,031	0,00409	0,44886
ENSGALT00000000318	ENSGALG00000000239	LOC101748951	27	1516164	0,502	0,00904	0,56252
ENSGALT00000002026	ENSGALG00000001330	ATP5G1	27	3472836	0,620	0,00262	0,36586
ENSGALT00000031321	-	LOC420043	27	4223822	0,904	0,00062	0,20712
ENSGALT00000021906	-	RX1	28	946749	0,692	0,00140	0,29811
NM_001081504	ENSGALG00000021647	NRTN	28	1117972	0,612	0,00806	0,54899
NM_001079751	ENSGALG00000001101	MBD3	28	1954898	-0,537	0,00116	0,27062
ENSGALT00000004080	ENSGALG00000002591	POLR2E	28	2402830	0,652	0,00728	0,53679
ENSGALT00000017943	ENSGALG00000028268	LOC417068	16_random	11985	1,341	7,13E-05	0,05807
ENSGALT00000000460	ENSGALG00000029093	MR1	16_random	25465	2,578	4,28E-05	0,04461
ENSGALT00000009357	ENSGALG00000026514	-	16_random	48484	1,421	1,36E-06	0,00450
ENSGALT00000000236	ENSGALG00000027445	LOC417056	16_random	116759	0,948	0,00380	0,44759
NM_001031473	ENSGALG00000002607	Y-Lec1	16_random	136130	0,721	0,00402	0,44886
ENSGALT00000040603	-	Q7LZS7	18_random	7186	-0,652	0,00551	0,49259
ENSGALT00000008254	ENSGALG00000005141	HMCN1	8_random	220237	-0,642	0,00088	0,23840
ENSGALT00000016693	ENSGALG00000010260	AQP2	E22C19W28_E50C23	798010	0,853	0,00135	0,29811
ENSGALT00000029908	-	LOC430551	E22C19W28_E50C23_ random	56090	0,731	0,00868	0,56166
ENSGALT00000041319	-	LOC426064	E64_random	24789	0,675	0,00821	0,54993
ENSGALT00000016523	ENSGALG00000010161	DMRT3	Z	26801250	0,661	0,00411	0,44886
ENSGALT00000038365	ENSGALG00000023317	-	Z	28367706	-0,599	0,00164	0,30823
ENSGALT00000038210	-	SMC5	Z	34628291	0,614	0,00782	0,54803
ENSGALT00000011005	ENSGALG00000006803	C20orf134	Z	44144955	-0,722	4,34E-05	0,04461
ENSGALT00000000313	ENSGALG00000000234	EPB41L4A	Z	45393662	-0,739	0,00061	0,20712

ENSGALT00000037857	-	HISPPD1	Z	48940520	-1,486	0,00568	0,49969
NM_001097534	ENSGALG00000015372	ATP5I	Z	52365085	0,975	0,00450	0,45369
ENSGALT00000037645	ENSGALG00000023087	-	Z	62581545	-0,527	0,00796	0,54803
