# Supplementary material for: Domestication and tameness: brain gene expression in red junglefowl selected for less fear of humans suggests effects on reproduction and immunology
Source: R Soc Open Sci. 2016 Aug 3;3(8):160033. doi: 10.1098/rsos.160033 (PMC5108935; doi:10.1098/rsos.160033)
Supplement: Table S2. Primer sequences used in qPCR verification of probe expression in P0 and S5 birds. Bird weight data. Bird weights at hatch, at 112, and 200 days for parental generation P0 and selection generation S5 [file rsos160033supp2.pdf]

Table S2. Primer sequences used in qPCR verification of probe expression in P0 and S5 birds.

Gene	Direction	Sequence
SPAG4	Forward	5'-AAAATCCGGCCCTCTTGTGT-3'
SPAG4	Reverse	5'-TCCAGTCCAAAGACAGCGAC-3'
GABRR1	Forward	5'-AGCAGGGCAGTAGTCCAATC-3'
GABRR1	Reverse	5'-TGGTCGTCTATTCGCAGCAG-3'
GPR112	Forward	5'-GTGAGTGCCAGCGTTGAAAA-3'
GPR112	Reverse	5'-AACCACCCAGTCCATCGTTC-3'
OTOR	Forward	5'-AGCTGTGTGCTGATGACGAC-3'
OTOR	Reverse	5'-CTCCATAAACACTTCCAGCCC-3'
Pol II for	Forward	5'-AAGGAGCCGCAGGTCTAC-3'
Pol II rev	Reverse	5'-CTTGCTCTTTGCCGTCATAC-3'
TBP for	Forward	5'-TAGCCCGATGATGCCGTAT-3'
TBP rev	Reverse	5'-GTTCCCTGTGTCGCTTGC-3'
