# Supplementary material for: Domestication and tameness: brain gene expression in red junglefowl selected for less fear of humans suggests effects on reproduction and immunology
Source: R Soc Open Sci. 2016 Aug 3;3(8):160033. doi: 10.1098/rsos.160033 (PMC5108935; doi:10.1098/rsos.160033)
Supplement: Bird Weight data [file rsos160033supp3.pdf]

ID Selection	Generation	Sex(M=Male; F=Female)	Selection	Weight(g) Hatch	Weight (g) day 8	Weight (g) day 46	Weight (g) day 112	Weight(g) day 200
45001	P0	F	Intermediate	27,0	40,3	254	862,7	795
45002	P0	M	High	31,2	42,2	247,0	873,9	925
45003	P0	F	Intermediate	28,2	47,7	225.5	812,2	782
45004	P0	M	Intermediate	25,0	37,4	255.5	903,3	
45006	P0	M	High	26,3	42,0	300.5	976,6	1184
45007	P0	F	Intermediate	28,1	35,2	210.5	845,1	890,5
45008	P0	F	Low	25,4	38,2	241	808,4	679
45009	P0	M	Intermediate	27,4	37,9	211	753,6	1100
45012	P0	F	Low	29,2	43,3	263.5	893,8	1048,5
45016	P0	F	Intermediate	26,5	31,7	189.5	744,4	767,5
45017	P0	F	Intermediate	28,9	41,3	203,0	664,6	763
45018	P0	M	Low	25,0	35,2	262.5	1011,1	1120
45020	P0	M	High	32,1	40,9	229,0	694,8	1359
45022	P0	F	High	27,2	37,6	232	735,7	865
45023	P0	F	Intermediate	26,2	39,0	310	1092,6	
45024	P0	M	Low	29,4	42,4	238,0	829,7	949
45025	P0	F	Intermediate	28,3	37,2	217	731,9	892
45027	P0	F	Intermediate	24,8	37,8	207	736,9	639
45029	P0	M	Intermediate	25,6	39,3	279.5	981,2	1278
45030	P0	F	Intermediate	34,9	48,5	277,0	809,1	929
45031	P0	M	Intermediate	29,2	43,8	268,0	821,5	1075
45032	P0	M	High	28,4	37,4	279	922,8	1133
45033	P0	M	Intermediate	25,6	35,7	174.5	594,9	1004
45035	P0	F	Intermediate	27,7	38,8	223.5	663,7	717
45037	P0	F	Intermediate	23,6	33,4	192	823,5	1016
45039	P0	F	Intermediate	27,5	40,3	259,0	994,4	696
45041	P0	F	High	22,8	30,3	227	655,8	905
45043	P0	F	Low	26,3	42,9	265	722	
45044	P0	M	High	27,1	38,9	242	898,4	965
45045	P0	M	Low	26,2	40,5	249,0	807,6	1116
45048	P0	M	High	25,0	35,0	212,0	734,5	1192
45049	P0	F	Low	27,0	42,1	229	680,8	718
45050	P0	F	Intermediate	27,0	42,3	244,0	745,3	852
45051	P0	M	Intermediate	26,6	36,4	192.5	648,7	1069
45052	P0	M	Low	25,6	41,6	237	783,7	1248
45053	P0	F	High	27,4	38,6	249	811,1	779
45055	P0	M	Intermediate	25,7	35,9	253	895,5	1260
45056	P0	M	Low	24,8	38,5	248.5	789,6	1124
45058	P0	F	High	26,2	40,7	234	717,2	845,5
45060	P0	F	High	33,5	49,4	277,0	933,2	819
45061	P0	M	High	28,2	42,9	239	755,8	1453
45062	P0	M	Low	24,9	34,2	249	888,7	1219
45063	P0	F	Low	24,8	38,3	229.5	685,3	981
45064	P0	F	Low	26,7	36,8	212,0	736,0	777
45066	P0	F	Intermediate	30,9	48,2	269,0	1022,9	970
45067	P0	M	High	27,9	37,0	191	688,3	1227
45068	P0	F	Intermediate	24,5	33,5	216,0	630,6	979
45069	P0	F	High	28,7	44,0	251	899,3	780

45070	P0	F	Intermediate	26,9	40,8	261	862,3	787
45071	P0	M	Intermediate	27,3	40,2	279	943,4	
45074	P0	F	Intermediate	24,2	35,1	237.5	761,0	749,5
45075	P0	F	Low	25,6	41,0	239.5	700,4	895,5
45076	P0	M	High	28,0	42,5	260,0	806,6	1180
45077	P0	M	Intermediate	31,0	44,6	285	827,1	1167
45081	P0	M	Intermediate	29,9	34,8	204	847,4	
45082	P0	M	High	26,7	39,6	237	706,3	1201
45083	P0	F	Low	26,0	39,4	264.5	769,6	819
45084	P0	F	Intermediate	30,0	39,6	212	697	972
45085	P0	F	High	26,9	39,0	238	767,6	930,5
45086	P0	F	Intermediate	25,3	35,9	214.5	845,4	906,5
45087	P0	F	High	24,4	39,3	225.5	774,2	841
45088	P0	M	Low	29,6	44,4	252.5	984,3	1111
45089	P0	M	Intermediate	25,2	41,5	224.5	639,9	1186
45090	P0	F	Low	26,6	35,0	273	1023,5	901
45091	P0	F	Intermediate	27,2	38,5	257,0	903,0	828,5
45093	P0	M	Intermediate	24,0	34,9	236,0	896,3	1378
45094	P0	M	Low	24,9	38,0	239.5	747,8	1210
45097	P0	F	High	25,5	35,6	245,0	826,3	904
45099	P0	M	Low	28,1	40,9	259.5	997,9	1190
45101	P0	F	Low	26,7		145.5	585,7	
45102	P0	M	Low	27,5	39,3	284	918,6	1182
45104	P0	M	Intermediate	27,4	39,8	259.5	935,5	
45105	P0	M	Intermediate	31,5	43,7	237	860,9	1100
45106	P0	F	High	27,9	38,2	201	735,1	1001,5
